# Controlling Capillary Flow Rate on Lateral Flow Test Substrates by Tape

**DOI:** 10.3390/mi12050562

**Published:** 2021-05-16

**Authors:** Zhiqing Xiao, Yuqian Yang, Xingwei Zhang, Weijin Guo

**Affiliations:** 1 Department of Biomedical Engineering, Shantou Univeristy, Shantou 515063, China; 19zqxiao@stu.edu.cn (Z.X.); 18yqyang3@stu.edu.cn (Y.Y.); 2 Department of Mechatronic Engineering, Shantou Univeristy, Shantou 515063, China; zhangxw@stu.edu.cn

**Keywords:** capillary flow rate, synthetic paper, nitrocellulose, volume capacity, lateral flow tests, tape

## Abstract

Controlling capillary flow rate of sample liquid is of high interest for lateral flow tests, since the flow rate can affect the dissolution and mixing of the immunoreagents and the efficiency of immunoreactions. Here we develop a facile method to adjust the capillary flow rate on lateral flow test substrates by using tape to cover the surface of substrates. We test this method on the traditional lateral flow test substrate—nitrocellulose and a novel lateral flow test substrate—synthetic paper, which is a porous media made by interlocked off-stoichiometry thiol-ene (OSTE) micropillars. We found that after the surface was covered by tape, the average flow rate decreased to 61% of the original flow rate on nitrocellulose, while the average flow rate increased to at least 320% of the original flow rate on synthetic paper. More interesting, besides the increase of flow rate, the volume capacity of synthetic paper also increases after covered by tape. Furthermore, we investigated the influence of length and position of tape on the capillary flow rate for nitrocellulose. A longer tape will lead to a smaller flow rate. The influence of tape of same length on the flow rate is bigger when the tape is placed closer to the loading pad. These results can help in the flow rate control on lateral flow test substrates, and potentially improve the performance of lateral flow tests.

## 1. Introduction

Lateral flow tests utilize capillary force to pump the sample liquid. Sample liquid will flow through the conjugate pad, and reach test line and control line. During this process, sample liquid will dissolve the immunoreagents on the conjugate pad and mix with them. Then the mixture will react with the antibodies on the test line and control line. Sample flow rate will affect the efficiency of all these steps including dissolution, mixing and reaction. Usually the liquid flow on a porous lateral flow test strip follows the Washburn Equation [1,2], of which the flow rate is the biggest at the beginning, and then gradually decreases with time. Many methods have been developed to obtain a proper flow rate by either decelerate or accelerate the sample liquid flow. For some cases, the sample liquid flow needs to be decelerated to ensure complete dissolution, efficient mixing, or thorough reaction. For some cases, the sample liquid flow needs to be accelerated to ensure fast readout or avoid the evaporation of sample liquid during the test. In addition, different assays need different reaction time depending on their specific binding kinetics [3]. People have tried to delay the capillary flow rate for paper microfluidics by designing 2D paper networks [4,5], utilizing dissolvable sugar or bridges [6,7], magnetic timing valves [8], or an absorbent pad-based shunt [9]. Some other methods have been applied to enhance the capillary flow by designing multilayer paper microfluidic devices [10,11,12,13]. Capillary flow can be also accelerated by using hollow channels [14], sandwiching paper channels by flexible films [15], or applying triboelectric effect [16]. Channels by razor crafting can be used to accelerate or decelerate the capillary flow on paper microfluidic devices [17]. However, majority of the methods mentioned above need multi-step assembly or complex fabrication process, which is not compatible with the laminating technology widely used in the massive manufacturing of lateral flow test strips. In this work, we come up with a facile method to adjust the capillary flow rate by taping the surface of lateral flow test substrates. We test two substrates: nitrocellulose and synthetic paper. Nitrocellulose has been widely used as the substrate for lateral flow tests [18,19,20]. The advantages of nitrocellulose include low cost and high surface area, which makes it suitable for colorimetric assays. However, the microstructure of nitrocellulose is not uniform, and its pore size has a big variation, which results in its low performance in repeatability. In addition, nitrocellulose has strong autofluorescence, and thereafter big signal to noise ratio when applying fluorescent assays on it. Synthetic paper is a novel lateral flow test substrate, fabricated by multi-directional lithography of OSTE [21,22,23]. Synthetic paper is transparent, and has low autofluorescence and uniform microstructure. In addition, there are free thiol groups on the surface of synthetic paper, which can facilitate efficient bonding of immunoreagents. A comparison was conducted between nitrocellulose and synthetic paper in terms of microstructure, autofluorescence and surface chemistry when they are used as the substrates of fluorescent protein microarray [24]. Synthetic paper was proved to be a good substrate for fluorescent bioassays [22,24]. Here, we try to control the capillary flow rates on these two substrates by taping their surface, compare the experimental results, and briefly discuss the reasons behind the influence of tape.

## 2. Materials and Methods

**Figure 1:**
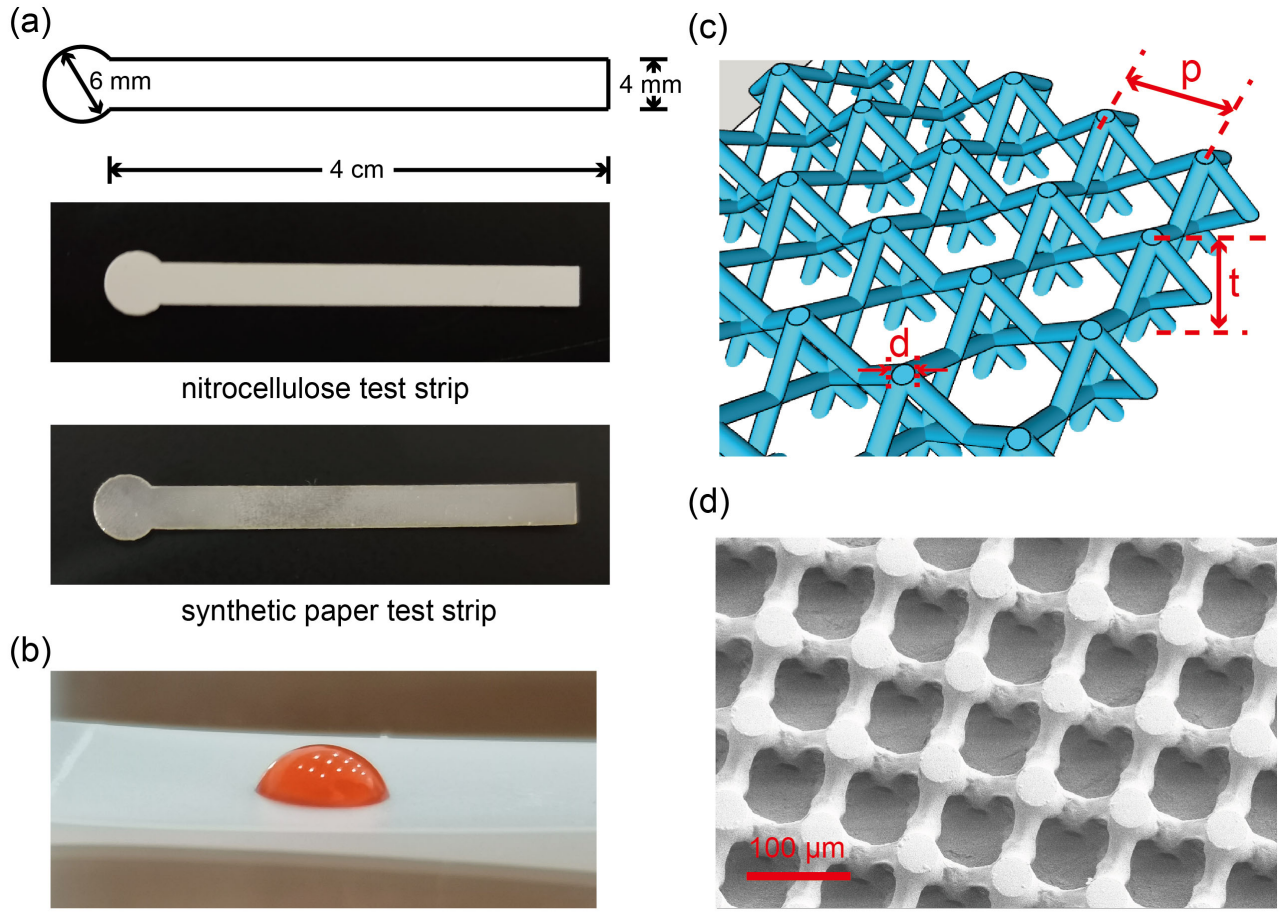
The nitrocellulose and synthetic paper test strips. (**a**) shows the schematic of the test strip, which consists of a loading pad (circle in the left) and a fluidic channel (rectangle in the right), and a picture of a nitrocellulose test strip and a picture of a synthetic paper test strip. (**b**) shows a drop of DI water with red food dye on the adhesive side of tape. (**c**) is a schematic of synthetic paper: d indicates the diameter of the micropillar, p indicates the pitch distance, and t indicates the thickness of synthetic paper. (**d**) is a SEM picture of synthetic paper. (**c**) is reprinted (adapted) from [24], Copyright (2020), with permission from Elsevier.

The nitrocellulose membrane (Hi-Flow Plus HF075) was purchased from Merck Millipore (Darmstadt, Germany). The thickness of nitrocellulose membrane was measured by a thickness micrometer (Qifeng, Linyi, China). Synthetic paper was fabricated by OSTE lithography [24]. Briefly, we fabricated a big piece of synthetic paper, then cut it to the desired shape. Both of nitrocellulose and synthetic paper test strips were cut by a laser cutter (Thunderlaser, China). The design of the test strip was by the software LaserMaker (Thunderlaser, China), and was identical for nitrocellulose and synthetic paper, as shown in Figure 1a. After cutting, we did the hydrophilic treatment for synthetic paper to facilitate capillary filling because the natural surface of synthetic paper was slightly hydrophobic. In detail, we put the synthetic paper test strips into Tween 20 solution (Macklin, Shanghai, China; 0.05% in DI water) and put the solution in a vacuum chamber to let Tween 20 solution penetrate into synthetic paper. After that, we took the synthetic paper test strips out and let them dry at room temperature before use. No hydrophilic treatment was needed for nitrocellulose since it underwent similar hydrophilic treatment during the manufacturing process. The tape used in this experiment was Scotch^®^ Magic^TM^ Tape from 3M (USA). We used a knife to cut it, and covered the surface of nitrocellulose or synthetic paper. The contact angle of water on the tape was measured by a contact goniometer (SDC-200S, Sindin, Dongguan, China). The surface of adhesive side of the tape was neutral, with a contact angle of 88.8°, shown in Figure 1b. The microstructure of synthetic paper (shown in Figure 1c, d) is: diameter *d* = 50 m, pitch distance *p* = 100 m, thickness *t* = 100 m. The test strips were attached to a glass slide (2.5 cm × 7.5 cm) by double sided tape (3M, USA), and placed horizontally. After the preparation of test strips, we dropped the DI water with red food dye (weight ratio as 1.25%) onto the loading pad by a pipette, and used a digital camera (EOS RP, Canon, Japan) to record the experimental video for data analysis. The data analysis was done by Video Analysis and Modeling Tool Tracker (https://physlets.org/tracker/, accessed on 18 January 2021). For nitrocellulose, every experiment was repeated three times. For synthetic paper, every experiment was repeated nine times. Because we found that in the experiments, the variation (error bar) of flow rates was big on synthetic paper. In order to have solid information about the fluidic behavior on synthetic paper, we repeated every experiment nine times. The big variation on synthetic paper was mainly caused by uneven imbibition, which can be the result of inhomogeneity in surface hydrophilicity.

## 3. Results and Discussion

**Figure 2:**
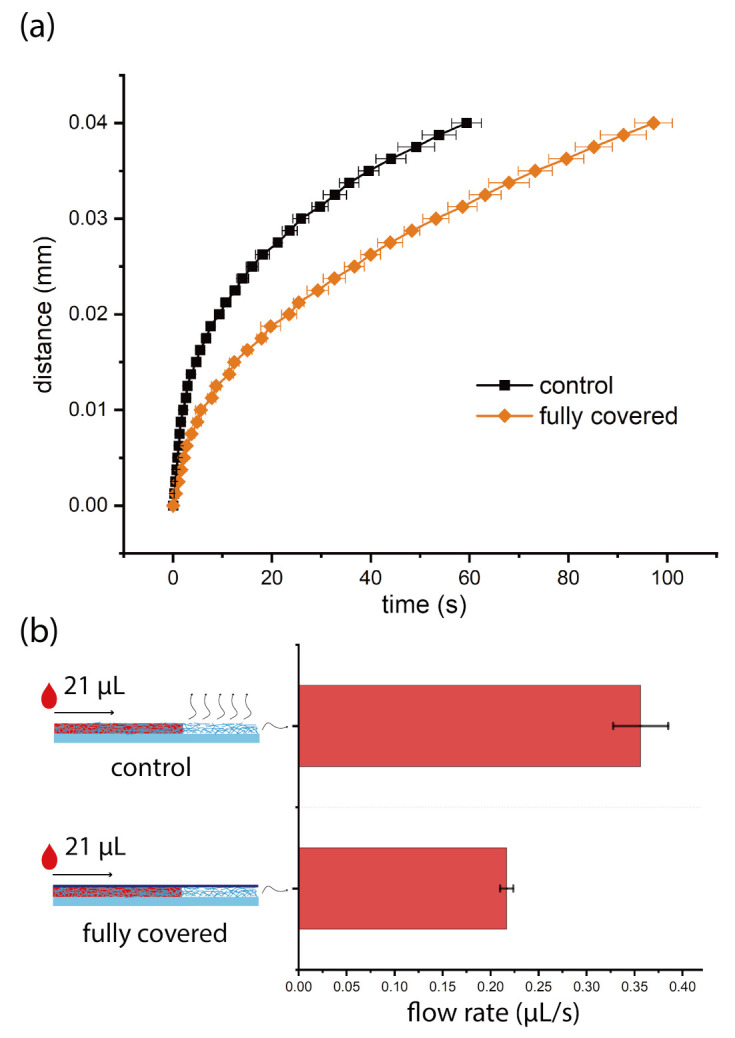
The flow behavior of DI water with red color dye on nitrocellulose test strips with or without tape. (**a**) is the time-distance plot: black solid dots are from the control group, and the orange solid dots are from the group of surface fully covered by tape. (**b**) shows the average flow rates: the left shows the schematic of liquid imbibition in nitrocellulose test strips with or without tape, the curvy arrows indicate the air propelled out from nitrocellulose, the right shows the comparison of average flow rates. On nitrocellulose test strips, every experiment is repeated three times.

**Figure 3:**
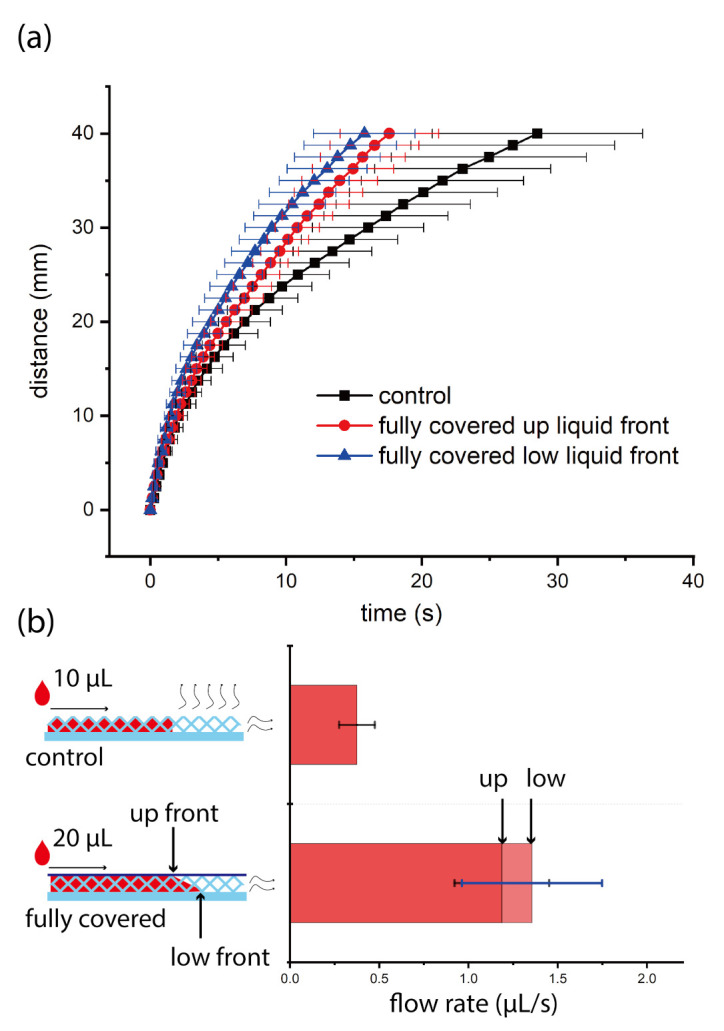
The flow behavior of DI water with red color dye on synthetic paper test strips with or without tape. (**a**) is the time-distance plot: black solid dots are from the control group, the red and blue solid dots are from the group of surface fully covered by tape. The red solid dots show the time-distance relation of the up liquid front and the blue solid dots show the time-distance relation of the low liquid front. (**b**) shows the average flow rates: the left shows the schematic of liquid imbibition in the synthetic paper test strips with or without tape, the curvy arrows indicate the air propelled out from synthetic paper, the right shows the comparison of average flow rates. On synthetic paper test strips, every experiment is repeated nine times. There are two liquid imbibition front lines observed on synthetic paper (7 out of 9 experiments). ’up front’ indicates the liquid front line attaching the surface of tape, and ’low front’ indicates the liquid front line attaching the bottom surface of synthetic paper. The arrow ’up’ in the right picture shows the average flow rate when we assume the imbibition liquid front is even and calculate the average flow rate using the time when ’up’ liquid front reaches the end point, and the arrow ’low’ in the right picture shows the average flow rate when we assume the imbibition liquid front is even and calculate the average flow rate when ’low’ liquid front reaches the end point.

We investigated the flow behavior of water on nitrocellulose and synthetic paper test strips with or without tape. Noticeably, the volume capacity (the volume of water absorbed by the test strips, more details can be found in Appendix) of nitrocellulose did not change after the surface was covered by tape (in Figure 2b). However, the volume capacity of synthetic paper increased after the surface was covered by tape (in Figure 3b). We think it was caused by the unique 3D topography of synthetic paper, the top liquid profile changed from the status shown as ’control’ to the status shown as ’fully covered’, as shown in Figure 3b. As a result, more water filled the voids and increased the volume capacity of synthetic paper. For nitrocellulose, there were no such voids or the voids between tape and its surface were too small to affect the volume capacity. Therefore, the volume capacity did not change for nitrocellulose after taping its surface. We studied the relation between time and imbibition distance on the test strips, and compared the results with and without tape, see Figure 2 and Figure 3. We found that for nitrocellulose, the flow rate decreased to 61% of the original value after the surface was covered by tape, while for synthetic paper the flow rate increased to at least 320% of the original value after the surface was taped. The flow rate increase on synthetic paper is the combined outcome of increase in both of pumping velocity and volume capacity. We think this contrast was mainly caused by the difference of pore size of two different substrates. The pore size of nitrocellulose was below 10 μm, and the dimension of nitrocellulose test strips was 144 μm × 4 mm × 40 mm, the pore size of synthetic paper was between 50 μm and 100 μm, and the dimension of synthetic paper test strips was 100 μm × 4 mm × 40 mm. During the liquid imbibition, liquid penetrated into the porous media for both of nitrocellulose and synthetic paper, and the air was propelled out. Usually, the air fluidic resistance in such a process can be neglected in large scale, but cannot be ignored in the micro scale [2]. For both of these two substrates, tape will block the air vents on the top surface, and air can only be propelled out from the air vents on the edges (the side edges and front edge). However, the pore size of synthetic paper was much larger than that of nitrocellulose, which meant that the air flow resistance (scale to r${}^{-3}$, r is the radius of the pore) of nitrocellulose was much larger than that of synthetic paper. As a result, for nitrocellulose, the increase of air fluidic resistance dominated the liquid imbibition process, so the flow rate decreased on nitrocellulose. For synthetic paper, the increase of air fluidic resistance had a much weaker effect on the flow rate. Moreover, after the surface of synthetic paper was covered by tape, the total inner surface area of the test strip increased. When the liquid penetrated into the synthetic paper covered by tape, it was easier to form more meniscus, which induced bigger Laplace pressure, and then increase of the pumping velocity. Besides adjusting the capillary flow rate on these two substrates, tape can also help to keep the surface clean during transportation or experiments since it can prevent the dust sediment on the surface.

In addition, we found that for the liquid flow on synthetic paper with tape, usually there were two different liquid front lines. One was faster, which was attaching the bottom surface of synthetic paper while the slow one was attaching the surface of tape, as shown in Figure 3b. We suspect that this was caused by different hydrophilicity of synthetic paper and tape. After hydrophilic treatment by Tween 20 solution, the surface of synthetic paper was more hydrophilic than tape (which was neutral). Therefore, the liquid advanced faster in the bottom and showed two liquid front lines with different positions. We calculated the average flow rate by equation: average flow rate = total volume/total pumping time. For the liquid flow on synthetic paper test strips with tape, we calculated two average flow rates. We used two total pumping times: one was the total time when the up front line reached the end point, and the other was the total time when the low front line reached the end point. The ’up’ flow rate was 320% and the ’low’ flow rate was 360% of the flow rate on synthetic paper test strips without tape. The actual flow rate should be between these two flow rates. So we concluded that the average flow rate increased to at least 320% of the original flow rate on synthetic paper after covering the surface by tape.

**Figure 4:**
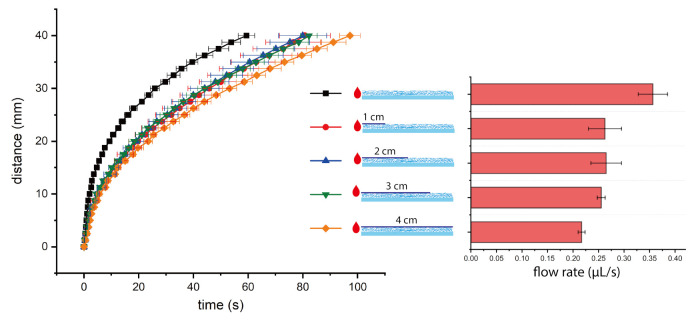
The time-distance plot of DI water flow on nitrocellulose test strips with tapes of different length (**left**), and the average flow rates of different groups (**right**). The tape started from the borderline between loading pad and fluidic channel.

**Figure 5:**
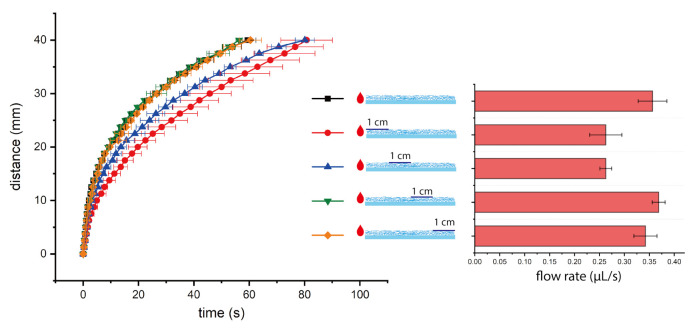
The time-distance plot of DI water flow on nitrocellulose test strips with tapes of same length but on different positions (**left**), and the average flow rates of different groups (**right**).

We further studied the influence of tape length and position on nitrocellulose test strips because of wider application of nitrocellulose as lateral flow test substrates. We tested four different tape lengths (1 cm, 2 cm, 3 cm, and 4 cm (fully covered)), and all the tapes started from the borderline between loading pad and fluidic channel. From the experimental results (shown in Figure 4), we found a trend that the longer tape will result in a lower flow rate. As we mentioned before, for nitrocellulose after taping the surface, the increase of air fluidic resistance played a major role in the liquid imbibition and decreased the flow rate. In addition, the tape also created some frictional drag during the capillary penetration. Therefore, the effect of a longer tape was more obvious and led to a lower flow rate. We also tested four different tape positions, all the tapes were of same length (1 cm), and started 0 cm, 1 cm, 2 cm, 3 cm from the borderline between loading pad and fluidic channel. As shown in Figure 5, we found a trend that the tape closer to the loading pad will have a bigger effect in decreasing flow rate. As we discussed, tape had an effect on lowering the flow rate for nitrocellulose. If tape of the same length was placed closer to the loading pad, the effect was more obvious since the tape in the upstream (closer to the loading pad) had a longer working time than the tape in the downstream. For example, for the comparison between tape starting from 0 cm and 3 cm from the borderline, the effect of tape (on lowering the flow rate) played a role from the beginning of capillary filling for tape starting from 0 cm, while the effect of tape only played a role in the end of capillary filling (when the liquid sample reached the point of 3 cm from the borderline). As a result, the tape closer to the loading pad had a bigger effect in decreasing flow rate. The influence was quite obvious for tapes 0 cm and 1 cm from the borderline, while it was not obvious for tapes 2 cm and 3 cm from the borderline. From the right bar chart in Figure 5, the error bars collapse for the group without tape, and the groups with tape 2 cm and 3 cm from the borderline. From the time-distance plot in the left of Figure 5, the curves from these three groups overlap with each other. Therefore, if the tape was placed far away from the loading pad, the influence on flow rate was insignificant. We notice that the variation of flow rates was quite high for some groups, and we think majority of the variation was from nitrocellulose itself. As shown in the right bar charts of Figure 4 and Figure 5, the flow rate variations of experimental groups (with tapes) were comparative or less than control group (without tapes). In addition, we did the Washburn Equation fitting for time-distance relations of all experiments on nitrocellulose and synthetic paper test strips (details can be found in Appendix), the flow behaviors on both substrates followed the Washburn Equation very well, while nitrocellulose group (R${}^{2}$ = 0.994) had a slightly higher R${}^{2}$ than synthetic paper group (R${}^{2}$ = 0.990).

We did a comparison of the performance of our method with other methods for capillary flow rate control, shown in Table 1. Among all the methods listed in the table, the performance of our method was in the medium range. Considering our method was simpler than the other methods, we can conclude the performance (versus fabrication complexity) of our method was very good. Nevertheless, there are some limitations of our work. We only tested our methods on two different lateral flow test substrates: nitrocellulose and synthetic paper, while there are some other substrate materials for lateral flow tests including cellulose and glass fiber sheet. Nitrocellulose from other companies other than Merck Millipore may have different performance when applying our method. For the outlook of our work, it will be very interesting to investigate the effect of our method on the performance of lateral flow immunoassays. However, careful experimental design is needed for such study since there are many other factors affecting the performance of immunoassays [3], which may cause some interference.

## 4. Conclusions

We develop an easy and fast way to adjust the capillary flow rate on the lateral flow test substrates: nitrocellulose and synthetic paper. After covering the surface with tape, the flow rate decreases on nitrocellulose but increases on synthetic paper. The capillary flow rate can also be adjusted by changing tape length or tape position on nitrocellulose. These results can help to obtain proper flow rates of sample liquid on lateral flow test substrates.

## Figures and Tables

**Table 1:** Comparison between our work and other methods for flow rate adjustment.

Reference	Working Principle	Lower Limit	Upper Limit
Songok and Toivakka [10]	utilizing gap between two surfaces	n.a.*	200%
Channon et al. [11]			16,900%
Channon et al. [13]			14,500%
Renault et al. [14]			700%
da Silva et al. [16]	utilizing triboelectric effect	55%	125%
Giokas et al. [17]	carving open channels on paper	69%	279%
This work	taping the surface of nitrocellulose	61%	n.a.*
	taping the surface of synthetic paper	n.a.*	320%

* n.a. is short for not applicable, which means the method is only for increasing the flow rate or decreasing the flow rate. The lower limit and upper limit are the values of ‘the flow rate adjusted/the original flow rate’.

**Figure A1:**
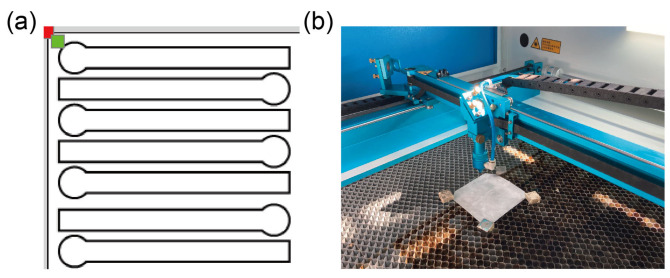
Cutting pattern design (**a**) and set up (**b**) of the laser cutter.

**Figure A2:**
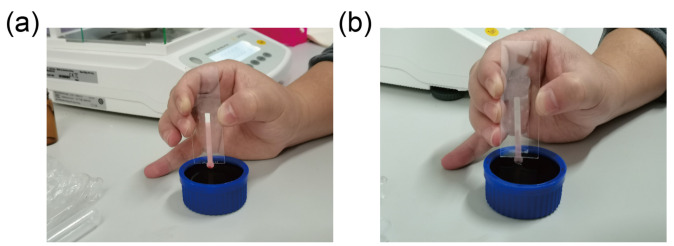
Water absorption experiments on nitrocellulose (**a**) and synthetic paper (**b**) test strips.

**Figure A3:**
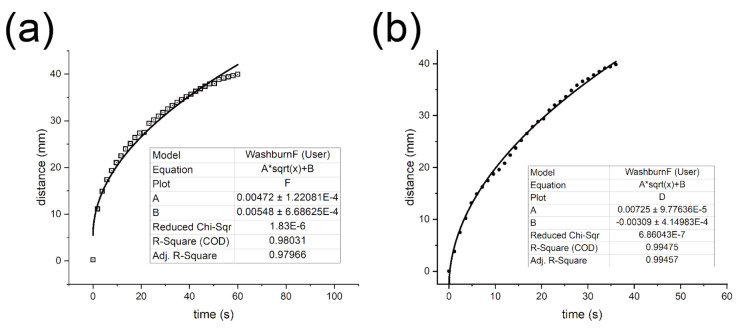
Washburn Equation fitting of flow behavior on nitrocellulose (**a**) and synthetic paper (**b**) test strips.

**Figure A4:**
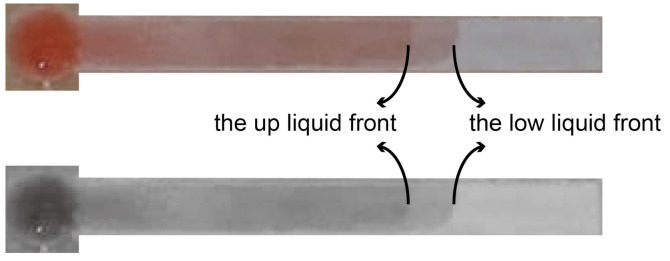
A synthetic paper test strip with liquid penetrating inside. A colorful picture in the top, and a gray picture (processed by ImageJ) in the bottom.

## Data Availability

The data is available per request to guoweijin@stu.edu.cn.

## Appendix A

### Appendix A.1. Fabrication of Nitrocellulose and Synthetic Paper Test Strips

At first we designed the cutting pattern (Figure A1a) by LaserMaker (Thunderlaser, Dongguan, China), then we sent it to the laser cutter for cutting. The nitrocellulose or synthetic paper was fixed on the substrate by magnets to ensure flatness of the nitrocellulose or synthetic paper piece, as shown in Figure A1b.

### Appendix A.2. Measurement of Water Absorption on Nitrocellulose and Synthetic Paper Test Strips

To investigate the influence of tape on the volume capacity of water absorbed on nitrocellulose and synthetic paper test strips, we conducted an experiment to measure the weight of water adsorbed on the test strips with or without tape. In detail, we prepared the test strips on glass slides and made sure that the loading pad did not contact with the glass slide. Then we dipped part of the loading pad in water solution with red color dye, and did the capillary filling experiments, as shown in Figure A2. We measured the weight of the test strips (with the glass slide together) before and after water absorption, and calculated the weight and volume of water absorbed in the test strips.

### Appendix A.3. Washburn Equation Fitting of Time-Distance Relation on Nitrocellulose and Synthetic Paper Test Strips

We used the software Origin 2021 (Massachusetts, USA) to do the Washburn Equation fitting of the time-distance relation on nitrocellulose and synthetic paper test strips to see if the flow behavior follows the Washburn Equation by checking the values of R${}^{2}$. The format of the fitting equation is Y = A * X${}^{1/2}$ + B (Y is the distance, X is the time). Figure A3 shows two examples of Washburn Equation fitting: (a) shows the fitting of a test of water filling on a nitrocellulose test strip, (b) shows the fitting of a test of water filling on a synthetic paper test strip.

### Appendix A.4. Two Fronts of Liquid Penetrating into a Synthetic Paper Test Strip

We noticed that for some synthetic paper test strips, there were two liquid fronts when we did the capillary flow experiments, one was attaching the bottom of synthetic paper, and the other one was attaching the surface of tape (shown in Figure A4). We can distinguish the different liquid fronts from the intensity of the color of a colorful experimental picture or a gray experimental picture processed by ImageJ (http://rsb.info.nih.gov/ij/, accessed on 12 May 2021).

## References

[1] Washburn E.W. (1921). The dynamics of capillary flow. Phys. Rev..

[2] Guo W., Hansson J., van der Wijngaart W. (2016). Capillary pumping independent of liquid sample viscosity. Langmuir.

[3] Squires T.M., Messinger R.J., Manalis S.R. (2008). Making it stick: Convection, reaction and diffusion in surface-based biosensors. Nat. Biotechnol..

[4] Fu E., Lutz B., Kauffman P., Yager P. (2010). Controlled reagent transport in disposable 2D paper networks. Lab Chip.

[5] Lutz B.R., Trinh P., Ball C., Fu E., Yager P. (2011). Two-dimensional paper networks: Programmable fluidic disconnects for multi-step processes in shaped paper. Lab Chip.

[6] Lutz B., Liang T., Fu E., Ramachandran S., Kauffman P., Yager P. (2013). Dissolvable fluidic time delays for programming multi-step assays in instrument-free paper diagnostics. Lab Chip.

[7] Houghtaling J., Liang T., Thiessen G., Fu E. (2013). Dissolvable bridges for manipulating fluid volumes in paper networks. Anal. Chem..

[8] Li X., Zwanenburg P., Liu X. (2013). Magnetic timing valves for fluid control in paper-based microfluidics. Lab Chip.

[9] Toley B.J., McKenzie B., Liang T., Buser J.R., Yager P., Fu E. (2013). Tunable-delay shunts for paper microfluidic devices. Anal. Chem..

[10] Songok J., Toivakka M. (2016). Enhancing capillary-driven flow for paper-based microfluidic channels. ACS Appl. Mater. Interfaces.

[11] Channon R.B., Nguyen M.P., Henry C.S., Dandy D.S. (2019). Multilayered microfluidic paper-based devices: Characterization, modeling, and perspectives. Anal. Chem..

[12] Schaumburg F., Berli C.L. (2019). Assessing the rapid flow in multilayer paper-based microfluidic devices. Microfluid. Nanofluidics.

[13] Channon R.B., Nguyen M.P., Scorzelli A.G., Henry E.M., Volckens J., Dandy D.S., Henry C.S. (2018). Rapid flow in multilayer microfluidic paper-based analytical devices. Lab Chip.

[14] Renault C., Li X., Fosdick S.E., Crooks R.M. (2013). Hollow-channel paper analytical devices. Anal. Chem..

[15] Jahanshahi-Anbuhi S., Chavan P., Sicard C., Leung V., Hossain S.Z., Pelton R., Brennan J.D., Filipe C.D. (2012). Creating fast flow channels in paper fluidic devices to control timing of sequential reactions. Lab Chip.

[16] da Silva E.T., Santhiago M., de Souza F.R., Coltro W.K., Kubota L.T. (2015). Triboelectric effect as a new strategy for sealing and controlling the flow in paper-based devices. Lab Chip.

[17] Giokas D.L., Tsogas G.Z., Vlessidis A.G. (2014). Programming fluid transport in paper-based microfluidic devices using razor-crafted open channels. Anal. Chem..

[18] Fenton E.M., Mascarenas M.R., López G.P., Sibbett S.S. (2009). Multiplex lateral-flow test strips fabricated by two-dimensional shaping. Acs Appl. Mater. Interfaces.

[19] Fridley G.E., Holstein C.A., Oza S.B., Yager P. (2013). The evolution of nitrocellulose as a material for bioassays. MRS Bull..

[20] Mansfield M.A. (2009). Nitrocellulose membranes for lateral flow immunoassays: A technical treatise. Lateral Flow Immunoassay.

[21] Hansson J., Yasuga H., Haraldsson T., van der Wijngaart W. (2016). Synthetic microfluidic paper: High surface area and high porosity polymer micropillar arrays. Lab Chip.

[22] Guo W., Hansson J., van der Wijngaart W. Synthetic microfluidic paper with superior fluorescent signal readout. Proceedings of the 23rd International Conference on Miniaturized Systems for Chemistry and Life Sciences (μTAS 2019).

[23] Guo W., Hansson J., van der Wijngaart W. (2020). Synthetic Paper Separates Plasma from Whole Blood with Low Protein Loss. Anal. Chem..

[24] Guo W., Vilaplana L., Hansson J., Marco M.P., van der Wijngaart W. (2020). Immunoassays on thiol-ene synthetic paper generate a superior fluorescence signal. Biosens. Bioelectron..

